# Feasibility of multi-class dental caries detection using deep learning–based smartphone images: a pilot prospective study

**DOI:** 10.3389/froh.2026.1805448

**Published:** 2026-05-08

**Authors:** Ji-Yun Kang, Hee-Kyung Kim

**Affiliations:** 1 Department of Convergence Medical Science, Graduate School of Medicine, Ajou University, Suwon, Republic of Korea; 2 Department of Prosthodontics, Institute of Oral Health Science, Ajou University School of Medicine, Suwon, Republic of Korea

**Keywords:** classification, deep learning, dental caries, detection algorithms, smartphone

## Abstract

**Aim:**

This pilot study evaluated the feasibility of multi-class dental caries detection using deep learning–based smartphone images.

**Method:**

Seventy adults provided six self-taken intraoral smartphone images in a non-clinical setting. Four dentists annotated each image based on the visual assessment of caries using binary (none vs. caries), four-class (none, initial, moderate, or advanced), and five-class (none, initial-1, initial-2, moderate, or advanced), according to two internationally recognized criteria (ICDAS-II and ADA CCS). A YOLOv6-L6 deep learning model was trained on an expert-labeled dataset, with model performance evaluated using sensitivity, specificity, precision, accuracy, F1-score, and mean average precision (mAP).

**Results:**

Intra-examiner reliability was substantial to almost perfect (κ = 0.765–0.952), whereas inter-examiner agreement was low to moderate (κ = 0.358–0.406). Across all classification schemes, the models achieved a sensitivity ≥86%, a specificity >97%, a precision >90%, and an accuracy >93%. F1-scores remained high (>88%), declining as the classes increased. The binary classification model demonstrated the highest mAP (65.2%), whereas the four- and five-class models showed progressively lower mAP values. Notably, the five-class model achieved the highest specificity (98.45%).

**Conclusion:**

This pilot study demonstrates the feasibility of smartphone-based deep learning as a supportive aid for caries detection and its potential role in patient-driven screening.

## Introduction

Dental caries is a highly prevalent and multifactorial disease characterized by a dynamic interplay between demineralization and remineralization of dental hard tissues, driven by bacterial acids produced through the fermentation of dietary sugars, particularly free sugars, which serve as the primary substrate for cariogenic bacteria and represent a key modifiable risk factor for caries development (1, 2). Early carious lesions typically manifest as subtle changes in enamel translucency or surface texture, such as white spot lesions, which are often imperceptible without controlled examination conditions such as air-drying and adequate lighting. Detection is further complicated by the anatomical inaccessibility of predilection sites such as proximal surfaces and occlusal fissures, as well as the inherent subjectivity of visual assessment, which renders early-stage diagnosis particularly challenging in routine clinical practice (3, 4). Visual inspection is the most commonly used method for screening caries on occlusal surfaces, typically considered the first-line diagnostic approach. Nevertheless, its accuracy may be constrained not only by the subjective judgment of clinicians but also by the requirement that patients present in person for examination (5). Bitewing radiographs are commonly used to detect proximal lesions. However, for occlusal caries, they exhibit low sensitivity (true-positive rate) for early-stage detection and cannot reliably differentiate between non-cavitated and cavitated lesions or between active and arrested lesions (6). Adjunctive tools such as laser fluorescence, electrical conductance, and quantitative light-induced fluorescence have shown good performance, especially in detecting early carious lesions. However, these techniques are often associated with reduced specificity (false-positive rate) and inconsistent reproducibility, particularly in clinical settings, owing to confounding factors such as staining, operator variability, and environmental conditions (7, 8).

In response to the clinical demand for reliable caries detection and assessment, visual scoring systems such as the International Caries Detection and Assessment System (ICDAS-II) and the American Dental Association Caries Classification System (ADA CCS) have been developed and continuously refined. ICDAS-II, proposed in 2005, examines tooth surface features and classifies the caries process using a digit scale ranging from 0 to 6, based on the severity of visual signs (9). ICDAS-II has demonstrated good reproducibility and validity in detecting and classifying caries lesions (9, 10), with strong correlations reported between ICDAS-II scores and histological lesion depth (11) Moreover, ICDAS-II was found to outperform bitewing radiography in the detection of occlusal caries (12). The ADA CCS, developed in 2008, focuses on clinical decision-making by categorizing carious lesions not only by severity but also by appropriate treatment strategies, making it advantageous for daily clinical practice (13). This system classifies the entire spectrum of caries into four stages—“sound”, “initial” (visually noncavitated), “moderate” (early cavitated), and “advanced” (deep cavitated)—corresponding to ICDAS codes 0, 1–2, 3–4, and 5–6, respectively (13).

Caries detection using clinical photographs captured with digital cameras has been explored previously. One study (14) reported that photographic caries lesion detection achieved accuracy comparable to on-site intraoral examinations, and that the photographer's professional status did not affect diagnostic outcomes. Another study (15) found that photographic assessment showed a higher sensitivity than visual inspection when validated against histological sections. Recently, artificial intelligence (AI) has been applied for detecting caries lesions from photographs, attracting increasing attention particularly since the COVID-19 pandemic, and its relevance extends well beyond this context. Access to dental care remains limited in many regions due to geographical, economic, and workforce-related barriers, underscoring the need for accessible, patient-driven screening tools that can facilitate early risk identification outside the clinical setting. Furthermore, diagnostic accuracy in visual caries assessment is known to vary considerably depending on clinician experience and training, contributing to inconsistent detection outcomes in routine practice. Standardized image-based assessment, supported by AI, has the potential to reduce this examiner-dependent variability by providing a reproducible and objective evaluation framework that is independent of the clinical setting and examiner expertise. In particular, Kühnisch et (16). reported over 90% accuracy when applying convolutional neural networks (CNNs) to single-tooth photographs. Similarly, Yoon et al. (17) developed a model that recognized tooth numbers with a mean average precision (mAP) of 88% and detected caries with an mAP of 77%.

Building on studies using conventional digital cameras, recent advances in mobile communication, digital imaging, and AI have positioned smartphone-acquired images as emerging resources in telehealthcare. Despite potential variability in zoom, angle, or lighting, improvements in smartphone cameras have markedly enhanced the reproducibility and diagnostic accuracy of clinical (18). Ou et al. (19) developed a multimodal fusion network integrating smartphone images with metadata for skin lesion diagnosis, achieving an accuracy of 76.8%. This finding highlights the broad potential of smartphone-based AI for medical imaging. Similar approaches have been extended to the oral cavity. For example, Lin et al. (20) proposed a CNN (HRNet) for oral cancer detection using smartphone photographs, reporting a sensitivity of 83.0%, a specificity of 96.6%, and a precision of 84.3%. More recently, Lamas-Lara et al. (21) evaluated smartphone-based detection of caries lesions against clinical visual diagnosis, demonstrating high sensitivity (90.2%), specificity (95.2%), and excellent inter-examiner agreement (κ > 0.935).

While previous studies have explored caries detection using clinical photographs captured by dental professionals under controlled conditions, the feasibility of multi-class caries staging from self-acquired smartphone images in a non-clinical setting remains largely unexplored. Most existing approaches have been limited to binary classification, without addressing lesion severity or the fine-grained differentiation of early-stage lesions. In this pilot study, we extend this line of research by evaluating whether smartphone images acquired by patients themselves, without professional assistance or standardized clinical equipment, can support stage-specific caries detection using deep learning. A key distinguishing feature of this study is the further subdivision of initial lesions into Initial-1 and Initial-2, directly addressing the challenge of early caries identification, which represents the most clinically critical yet diagnostically difficult stage. Collectively, this study aims to establish the groundwork for patient-driven, smartphone-based caries screening as an accessible adjunct to conventional clinical evaluation.

## Methodology

### Data collection

A total of 70 participants were recruited for this pilot prospective study between November 2023 and May 2024. Adults aged 20–70 years who were able to follow the smartphone imaging instructions and provide intraoral photographs were included, whereas individuals with edentulous dentition in either the maxillary or mandibular arch were excluded. Each participant took six intraoral smartphone photographs according to the imaging guidelines provided in this study: (1–3) frontal, right lateral, and left lateral views with the teeth in occlusion and the lips parted to capture the labial surfaces; and (4–6) anterior, right, and left mandibular views with the mouth open to expose as much of the tooth surface as possible, including the occlusal surfaces. Participants were instructed to use the camera's grid function to center the image for optimal alignment, maintain a consistent shooting distance, avoid using a flash, and use a front-facing camera (selfie mode). They were advised not to use the zoom function and to position the camera lens parallel to the tooth surface to ensure consistent angulation. To improve the visibility of early enamel changes, participants were additionally instructed to swallow saliva prior to image acquisition and to capture images under relatively dry intraoral conditions. Given the inherent difficulty of capturing posterior teeth in self-acquired intraoral photographs, participants were guided to adjust head position and retract the cheeks to enhance visualization of posterior regions. In addition, standardized imaging protocols were applied to reduce variability in angulation and framing across participants. If submitted images did not meet predefined quality criteria, including insufficient visualization of posterior areas, participants were required to repeat image acquisition for the corresponding view until acceptable quality was achieved. Photographs that were out of focus, exhibited excessive shadows or distortions, or failed to clearly depict intraoral anatomical structures were excluded from the study. This approach enabled quality control at the point of data collection, thereby improving consistency while preserving the real-world characteristics of self-acquired smartphone images. All images were anonymized and stored without identifying information, showing only the dentition of the participants. Detailed smartphone acquisition parameters, including imaging views and device distribution, are summarized in Supplementary Table S1. This study was conducted in accordance with the STARD-AI guidelines, and the completed checklist is provided in Supplementary Table S2.

### Annotation of lesions

**Figure 1:**
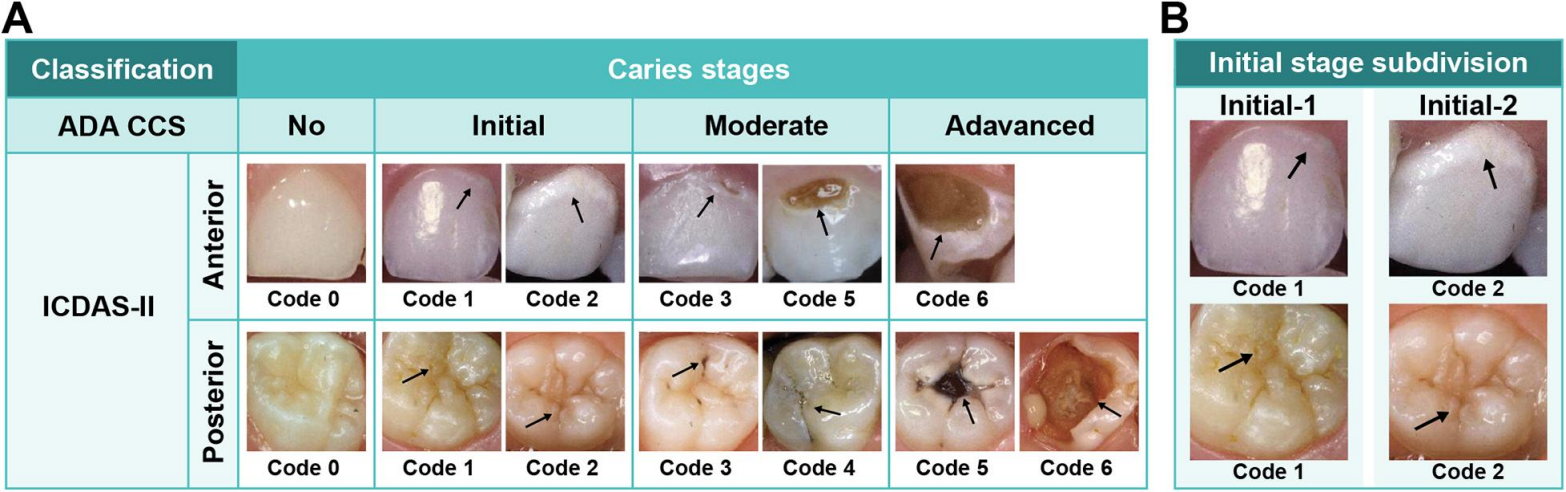
Mapping of caries classification systems and five-class subdivision. **(A)** Relationship between the American dental association caries classification system (ADA CCS) and the international caries detection and assessment system II (ICDAS-II) for anterior and posterior teeth across the stages of no, initial, moderate, and advanced caries. **(B)** Subdivision of the initial stage into Initial-1 (code 1) and Initial-2 (code 2) for finer differentiation of early enamel lesions. Used with permission of John Wiley & Sons, from pediatric dentistry: a clinical approach (34). Permission obtained through Copyright Clearance Center, Inc.

To ensure precision and consistency in dental caries scoring, the annotation process was conducted by four dentists, each with more than 5 years of clinical experience. The examiners were blinded to participants' identities. All four were calibrated and trained according to the ICDAS-II and ADA CCS examination protocols and manually annotated lesions based on three classification criteria derived from these two visual scoring systems: binary (none vs. caries), four-class (none, initial, moderate, or advanced), and five classes (none, initial-1, initial-2, moderate, or advanced). The four-class scheme followed the ADA CCS criteria, whereas the five-class scheme was adapted by incorporating ICDAS-II to subdivide the initial stage into initial-1 and initial-2 to evaluate the ability of the model to differentiate early caries. The correspondence between the ICDAS-II and ADA CCS systems, along with the five-class subdivision used in this study, is illustrated in Figure 1. During annotation, examiners differentiated carious lesions from visually similar conditions based on ICDAS-II and ADA CCS visual criteria, identifying caries by features such as white spot lesions, brownish discoloration, and surface cavitation. Surfaces showing findings inconsistent with these criteria, including restorations, fluorosis, enamel hypoplasia, or developmental anomalies, were classified as sound and excluded from caries labeling. When a single tooth surface displayed carious features corresponding to more than one severity level, the surface was classified according to the most severe lesion present, consistent with the clinical principle that treatment decisions are guided by the most advanced pathological finding.

**Figure 2:**
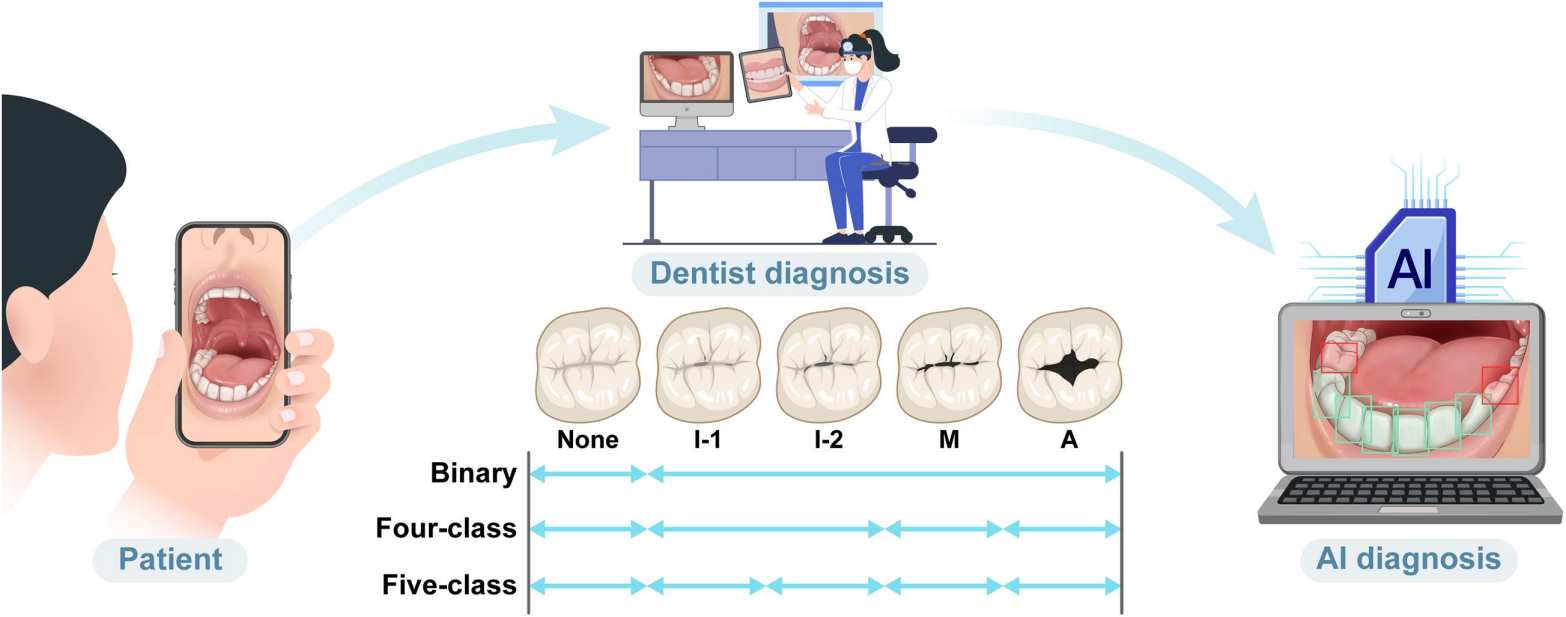
Schematic of the workflow for expert-labeled annotations and classification of smartphone-acquired intraoral images. Patients captured images using a smartphone, which were reviewed and annotated by dental experts based on three classification schemes: binary (none vs. caries), four-class (none, initial, moderate, or advanced), and five-class (none, initial-1, initial-2, moderate, or advanced). These annotations served as ground-truth data for training and evaluating the AI diagnostic model.

Each examiner assessed the same set of images on two separate occasions, spaced three weeks apart (22), to evaluate intra-examiner reliability. For consensus scoring, the results from both rounds (rounds 1 and 2) were aggregated by all examiners. Scores with ≥85% agreement were directly accepted as the consensus, whereas cases with lower agreement were collectively reviewed until consensus (≥85%) was reached. For each tooth surface, scores from all four examiners across both rounds were aggregated, yielding eight scores per surface. A consensus was directly accepted when at least seven of the eight scores (≥87.5%) were in agreement; surfaces with lower agreement were collectively reviewed by all examiners until the ≥85% threshold was reached. This consensus process was applied to each of the 4,932 tooth-surface visual instances extracted from the 420 images, ensuring that every instance was assigned a single, expert-verified ground-truth label. The finalized consensus scores served as ground-truth labels for training the deep learning model. As the model was trained exclusively on these expert-verified consensus labels, the differential diagnosis implicitly performed during annotation, whereby surfaces attributable to non-carious conditions were classified as sound, was inherently reflected in the model's learning targets, ensuring that only visually confirmed carious lesions served as positive training instances. Accuracy and consistency of caries detection were evaluated by assessing intra-examiner reliability with weighted Cohen's kappa (23) and inter-examiner agreement with Fleiss' kappa (24). According to the Landis and Koch rating scale, agreement was interpreted as follows: 0.01–0.20, null or minimal; 0.21–0.40, low; 0.41–0.60, moderate; 0.61–0.80, substantial-good; and 0.81–1.00, almost perfect (25). Figure 2 illustrates the workflow for expert-labeled classification of smartphone-acquired oral images.

### Deep learning model

**Figure 3:**
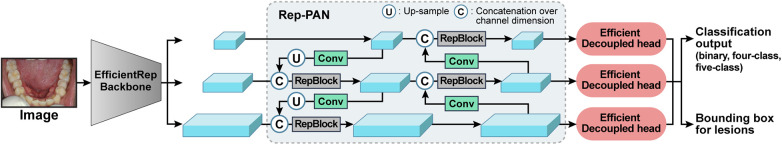
Schematic architecture of the YOLOv6-L6-based model for intraoral image analysis. The model utilizes a smartphone-acquired intraoral image as input and processes it through an EfficientRep backbone for feature extraction, followed by a Rep-PAN neck for multiscale feature aggregation using up-sampling **(U)**, convolution (Conv), and concatenation **(C)** operations. The output features are passed to efficient decoupled heads, which generate classification outputs (binary, four-class, or five-class) and lesion-level bounding box predictions.

In this pilot study, a CNN-based YOLOv6-L6 model was employed to investigate the feasibility of smartphone-based multi-class caries detection (Figure 3). The YOLOv6-L6 architecture consists of an EfficientRep backbone for feature extraction, a Rep-PAN neck for multiscale feature aggregation, and a decoupled detection head for classification and localization.

A total of 420 intraoral smartphone images were collected from 70 participants (six images per participant). To prevent data leakage, dataset splitting was first performed at the patient level, such that all images and corresponding teeth from a given participant were assigned exclusively to either the training, validation, or test set in an 8:1:1 ratio. For model development, the analytical unit was defined at multiple levels depending on the study stage. Following patient-level splitting, tooth surfaces visible in each image were annotated for caries status and treated as visual instances for model training. Although caries categories were not defined separately for buccal and occlusal surfaces, tooth surfaces captured under different viewing angles and imaging conditions were treated as complementary visual observations. As a result, a total of 4,932 tooth-surface visual instances were generated and used for model training. These instances represent repeated visual observations rather than independent biological samples. Table 1 summarizes the tooth-level distribution of caries categories across the training, validation, and test sets based on the five-class classification scheme (none, initial-1, initial-2, moderate, or advanced).

**Table 1:** Tooth-level distribution of caries categories across the training, validation, and test sets after patient-level dataset splitting.

Caries	Train set	Validation set	Test set
None	3,537	442	442
Initial-1	160	20	20
Initial-2	106	13	14
Moderate	86	11	11
Advanced	56	7	7
Total	3,945	493	494

After patient-level dataset splitting and data preparation, image preprocessing and data augmentation were performed using the Augmentor tool on the Superb platform (Superb AI Inc., Seoul, Republic of Korea), which operates on a command-line-based core toolkit. To enhance model generalizability and reduce overfitting, augmentation techniques were applied exclusively to the training set to simulate variations in angulation, framing, lighting, and image quality, including random rotation (≤15°), cropping, horizontal and vertical flipping, noise addition, color jitter, and cutout/random erasing (26). The model was trained with an input size of 1,280 × 1,280 using a stochastic gradient descent (SGD) optimizer (momentum = 0.937, weight decay = 0.0005), a cosine annealing scheduler, and 24 epochs. VariFocal Loss (VFL) (27) and the SCYLLA Intersection over Union (SIoU) loss function (28) were combined, and Task-Aligned Learning (TAL) (29) was implemented to improve the stability and convergence of label assignment. The validation and test datasets were used without augmentation to ensure unbiased performance evaluation under consistent imaging conditions. During validation and testing, images were resized to 1,280 × 1,280 and normalized to the 0–1 range. In summary, the preprocessing pipeline consisted of three steps: (1) image cleaning through quality-based exclusion and participant retakes at the data collection stage; (2) resizing to 1,280 × 1,280 and pixel normalization to the 0–1 range; and (3) data augmentation applied exclusively to the training set to enhance model generalizability. The detailed training configuration for the YOLOv6-L6-based dental caries detection model is summarized in Supplementary Table S3.

**Figure 4:**
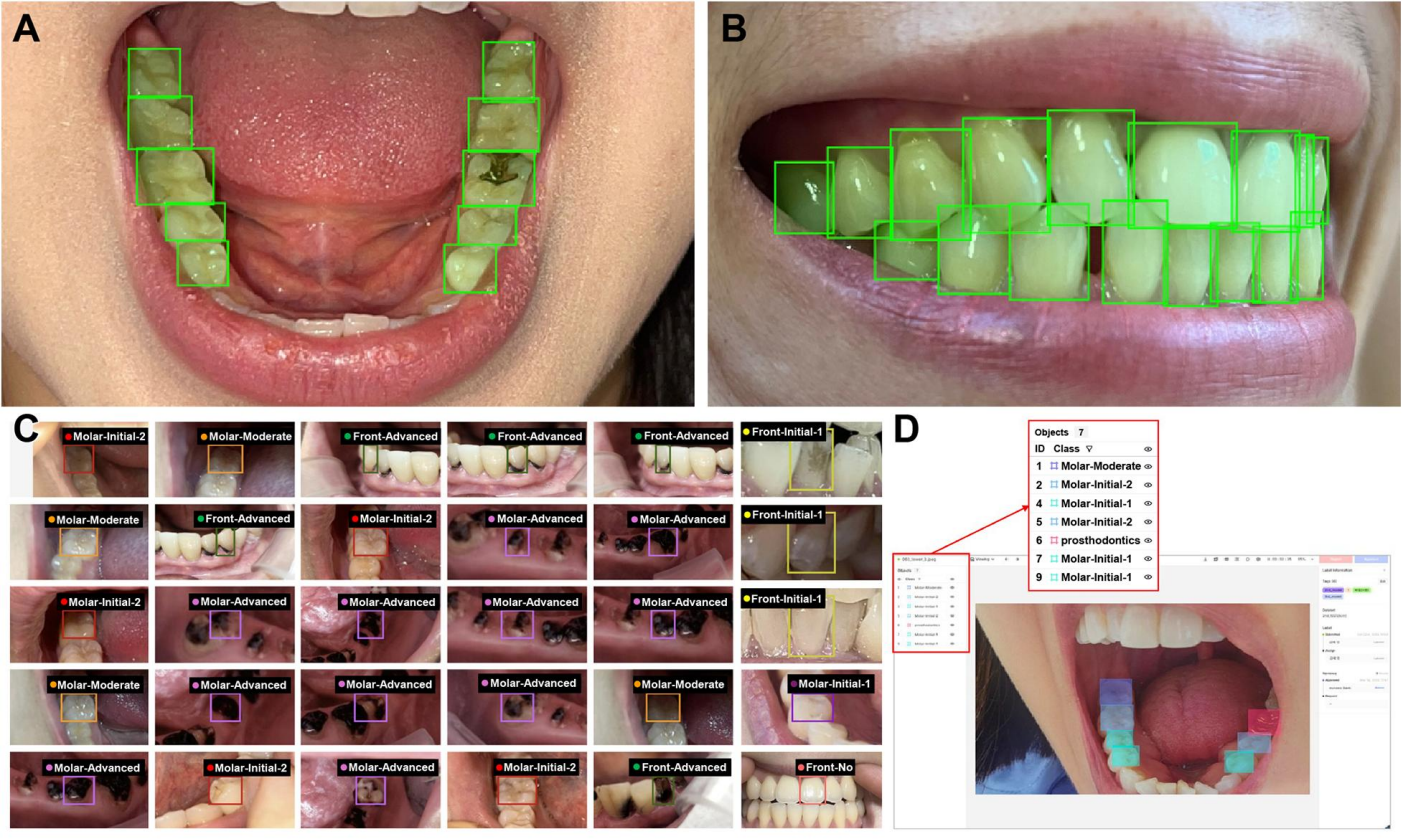
Tooth recognition and caries detection workflow using the YOLOv6-L6 model. **(A)** Recognition of the mandibular occlusal surface, where each tooth is localized within the lower arch for subsequent lesion detection. **(B)** Recognition of the labial surface in occlusion, enabling tooth-level localization from a frontal view. **(C)** Detection of individual teeth with caries lesions annotated according to class-specific severity. **(D)** Detection of all caries lesions present in a single individual, with bounding boxes and labels indicating each lesion by class.

**Figure 5:**
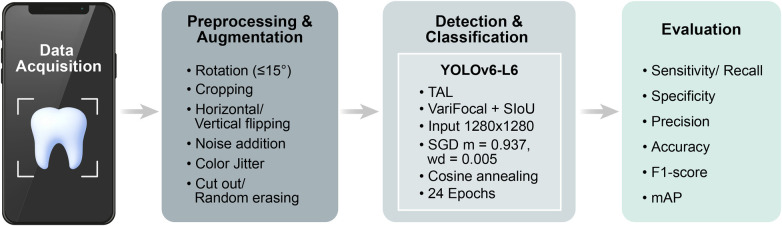
Deep learning pipeline for smartphone-based caries detection. The diagram illustrates the overall process from data acquisition to model evaluation using the YOLOv6-L6 framework with data augmentation and performance metrics including sensitivity/recall, specificity, precision, accuracy, F1-score, and mAP. TAL, task-aligned learning; SIoU, SCYLLA intersection over union; SGD, stochastic gradient descent; mAP, mean average precision.

To address class imbalance, underrepresented classes were merged prior to training based on clinical similarity. The YOLOv6-L6 model was initialized with weights pretrained on the COCO dataset and fine-tuned over 24 epochs using mini-batches of 16 images with corresponding expert-labeled annotations. A learning rate of 0.001 was used, along with data augmentation and loss-based optimization strategies, to mitigate overfitting. Carious lesions were detected using bounding boxes encoding their spatial locations and extents, and individual teeth were simultaneously localized to enable tooth-level spatial association within the oral cavity (Figures 4A,B). Detected lesions were subsequently assigned severity categories according to binary, four-class, or five-class classification schemes, with representative examples shown in Figure 4C and detection results from a single individual illustrated in Figure 4D. Model predictions were compared with consensus ground-truth annotations. For each classification scheme, model training was repeated seven times to assess robustness. Additional experiments were conducted using progressively larger subsets of the dataset (25%, 50%, 75%, and 100%), with performance monitored on the validation set during training and final evaluation performed on the independent test set. An overview of the complete pipeline, from data acquisition to model evaluation using the YOLOv6-L6 framework, is provided in Figure 5.

### Evaluation metrics

For performance evaluation, predictions corresponding to the same tooth appearing across multiple images were aggregated at the tooth level, ensuring that each tooth contributed only once to the final analysis. Each detection was uniquely matched to its corresponding ground-truth tooth label prior to metric calculation to prevent duplicate counting. Model predictions were compared with consensus ground-truth annotations. A detection was considered a true positive when the intersection over union (IoU) was ≥0.5, and non-maximum suppression (NMS) was applied with a threshold of 0.65. Duplicate detections assigned to the same tooth were counted as false positives, whereas carious teeth without any matched detections were considered false negatives. All performance metrics were computed on a per-tooth basis. Diagnostic performance was evaluated using confusion matrices constructed for the binary, four-class, and five-class classification schemes.

True positives (TPs) represent carious teeth correctly identified as carious, false positives (FPs) represent healthy teeth incorrectly predicted as carious, true negatives (TNs) represent healthy teeth correctly classified as non-carious, and false negatives (FNs) represent carious teeth missed by the model. Performance metrics derived from the confusion matrices included sensitivity [recall; TP/(TP + FN)], specificity [TN/(TN + FP)], precision [TP/(TP + FP)], accuracy [(TP + TN)/(TP + FP + TN + FN)], F1-score, and mean average precision (mAP). Sensitivity reflects the ability of the model to correctly identify carious teeth, whereas specificity indicates the ability to correctly classify non-carious teeth. Precision represents the proportion of teeth predicted as carious that were truly carious, and accuracy reflects the overall proportion of correct predictions. The F1-score, calculated as the harmonic mean of precision and recall, balances false positives and false negatives. The mAP was computed at an IoU threshold of 0.5 to jointly evaluate lesion localization and classification performance across categories.

### Statistical analysis

Data analyses were performed using the R software (version 4.2.3). Descriptive statistics were used to summarize the data, and the model performance metrics were reported with the corresponding values. Statistical significance was set at a threshold of *p* < 0.05. The *p*-values reported in Tables 2, 3 refer exclusively to the statistical significance of examiner agreement indices (weighted Cohen's kappa and Fleiss' kappa), confirming that the observed agreement levels were unlikely to have occurred by chance. These *p*-values do not apply to AI model performance evaluation, which was assessed solely using diagnostic metrics including sensitivity, specificity, precision, accuracy, F1-score, and mAP, without inferential statistical testing.

**Table 2:** Intra-examiner reliability: weighted Cohen's kappa coefficients for each examiner.

Rater	Weighted Cohen's Kappa	95% confidence interval	*p-*value
Examiner 1	0.952	0.932–1.000	*p* < 0.001
Examiner 2	0.913	0.880–0.946	*p* < 0.001
Examiner 3	0.866	0.823–0.908	*p* < 0.001
Examiner 4	0.765	0.726–0.805	*p* < 0.001

**Table 3:** Inter-examiner agreement: fleiss’ kappa for each diagnostic round.

Diagnostic round	Fleiss’ Kappa	*z-*value	*p-*value
First round	0.358	59.2	*p* < 0.001
Second round	0.406	65.8	*p* < 0.001

## Results

The intra-examiner reliability, assessed using weighted Cohen's kappa coefficients, ranged from 0.765 to 0.952 across the four examiners (Table 2). According to the Landis and Koch rating scale, examiner 1 [κ = 0.952, 95% confidence interval (CI) = 0.932–1.000] and examiner 2 (κ = 0.913, 95% CI = 0.880–0.946) demonstrated almost perfect agreement, examiner 3 (κ = 0.866, 95% CI = 0.823–0.908) showed substantial-good to almost perfect agreement, while examiner 4 (κ = 0.765, 95% CI = 0.726–0.805) achieved substantial-good agreement. Notably, examiner 1 had the longest clinical experience (30 years), whereas examiner 4 had the shortest clinical experience (6 years), suggesting that greater clinical experience was associated with higher intra-examiner reliability. Inter-examiner agreement, evaluated using Fleiss' kappa, increased from κ = 0.358 in the first round to κ = 0.406 in the second round (Table 3), corresponding to low and moderate agreement, respectively.

Weighted Cohen's κ values were calculated to assess inter-examiner agreement across the five caries categories (no, initial-1, initial-2, moderate, and advanced). As summarized in Table 4, κ values were lower for the no and initial categories, indicating greater diagnostic variability in early enamel lesions, whereas higher κ values for the moderate and advanced categories reflected stronger consensus.

**Table 4:** Per-class weighted Cohen's *κ* values for each examiner. Mean ± standard deviation (SD) represents the average *κ* across the four examiners for each class.

Class	Dentist 1	Dentist 2	Dentist 3	Dentist 4	Mean ± SD
No	0.623	0.641	0.694	0.112	0.52 ± 0.27
Initial-1	0.711	0.563	0.673	0.378	0.58 ± 0.15
Intiial-2	0.795	0.599	0.791	0.445	0.66 ± 0.17
Moderate	0.916	0.643	1.000	0.534	0.77 ± 0.22
Advanced	1.000	1.000	1.000	1.000	0.99 ± 0.21

**Figure 6:**
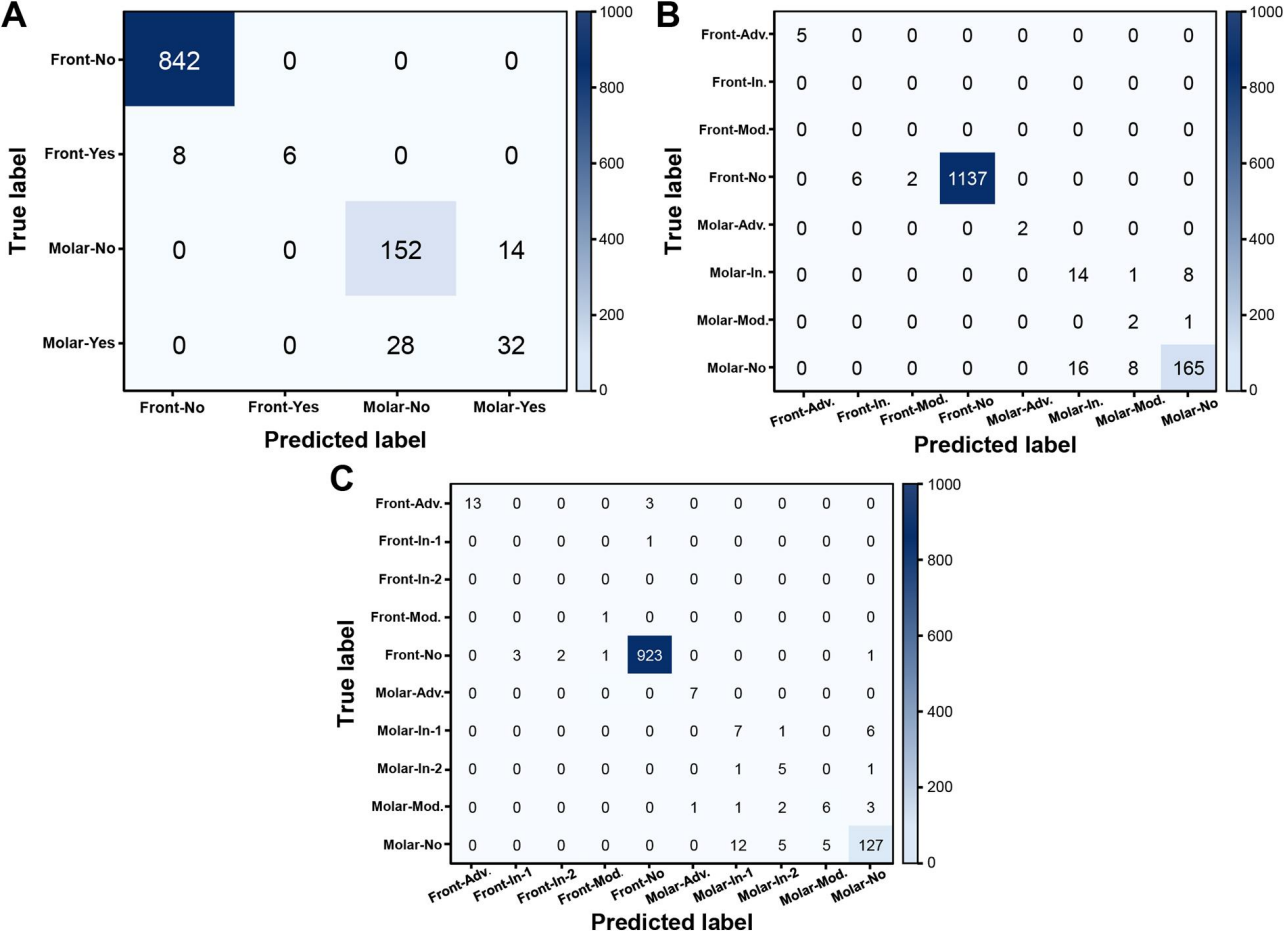
Confusion matrices of the classification models. Confusion matrices visualize the relationship between true and predicted labels for **(A)** binary, **(B)** four-class, and **(C)** five-class classification schemes. Correct predictions are represented along the diagonal, while off-diagonal entries indicate misclassifications. These matrices highlight cases where lesions were confused between adjacent severity levels, thereby providing insights into error patterns across different caries stages.

The confusion matrix shown in Figure 1 was obtained by applying the YOLOv6-L6 model to the test set. In the binary classification task (Figure 6A), the model effectively distinguished between caries and non-caries cases, with most samples correctly classified and only a small proportion misclassified. In the four-class classification task (Figure 6B), which included none, initial, moderate, and advanced stages, the model achieved high accuracy in the none category, although some misclassifications occurred between the adjacent severity levels, particularly between the initial and moderate levels. In the five-class classification task (Figure 6C), which further subdivided the initial stage into initial-1 and initial-2, although the model maintained strong performance in the none category, increased misclassification was observed across neighboring early-stage categories, such as initial-1 vs. initial-2 and initial-2 vs. moderate, indicating that the fine-grained classification of early caries stages is more challenging than binary and four-class tasks.

**Figure 7:**
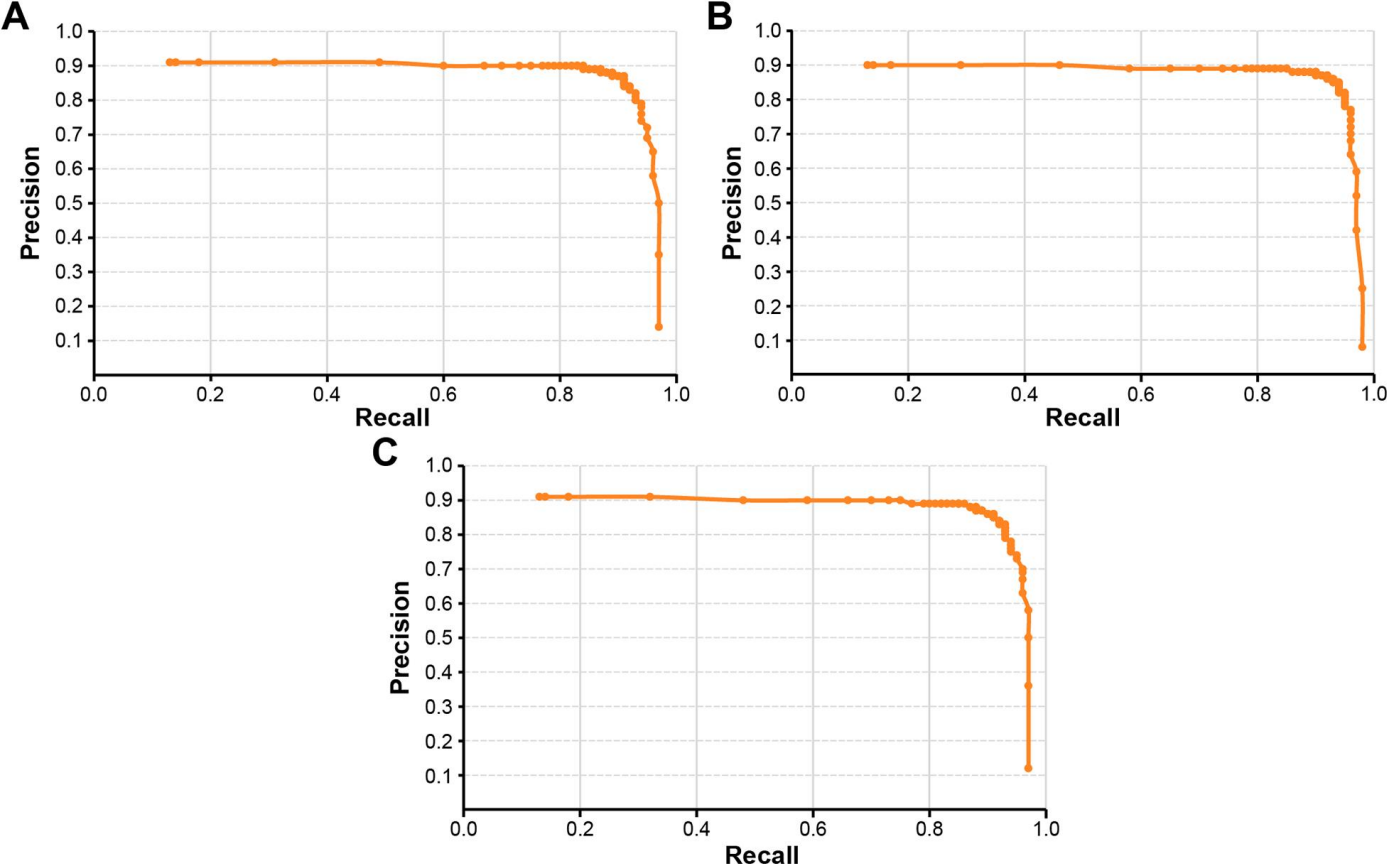
Precision-recall (PR) curves of the classification models. The PR curves illustrate the trade-off between precision and recall across varying decision thresholds for **(A)** binary, **(B)** four-class, and **(C)** five-class classification schemes. The area under each curve corresponds to the average precision, which was further used to calculate the mean average precision (mAP) values presented in Table 5.

The diagnostic performance of the YOLOv6-L6 model was further evaluated using the Precision-recall curves for the three classification schemes (Figure 7). The curves illustrate the trade-off between precision and recall across varying decision thresholds, with the area under each curve corresponding to the average precision. The binary classification scheme (Figure 7A) achieved the highest overall balance between precision and recall, whereas the four-class (Figure 7B) and five-class (Figure 7C) schemes demonstrated greater variability across thresholds, reflecting the increased difficulty in distinguishing between multiple severity levels. The mAP values derived from the curves are listed in Table 5.

**Table 5:** Overall performance metrics of the classification models. All values are reported as proportions between 0 and 1.

Metric	Binary	Four-class	Five-class
Optimal threshold	0.69	0.56	0.57
Sensitivity/Recall	0.87	0.86	0.86
Specificity	0.9671	0.9816	0.9845
Precision	0.90	0.92	0.90
Accuracy	0.9374	0.9440	0.9357
F1- score	0.8848	0.8892	0.8797
mAP (@0.5:0.95 IoU)	0.53	0.416	0.346
mAP (@0.5 IoU)	0.65	0.51	0.51

IoU, intersection over union.

Table 5 summarizes the overall performance of the three classification models. The binary classification model achieved a sensitivity of 87%, a specificity of 96.7%, a precision of 90%, and an accuracy of 93.7%, with an F1-score of 88.5%. The four-class model showed comparable sensitivity (86%) and higher specificity (98.2%) and precision (92%), with an accuracy of 94.4% and an F1-score of 88.9%. Similarly, the five-class model yielded a sensitivity of 86%, a specificity of 98.5%, a precision of 90%, and an accuracy of 93.6%, yielding an F1-score of 87.9%. Notably, the five-class model achieved the highest specificity (98.45%), effectively identifying non-carious cases.

In terms of detection performance, the binary model achieved the highest mAP value of 53.2% at Intersection over Union (IoU) thresholds of 0.5–0.95 and 65.2% at an IoU of 0.5. In contrast, the four- and five-class models exhibited progressively lower mAP values (41.6% and 34.6% at IoU = 0.5–0.95, and 51.0% and 45.0% at IoU = 0.5, respectively). These findings indicate that while the multi-class models maintained high diagnostic accuracy, their overall detection performance declined as the classification scheme became more granular. To further evaluate class-level performance, precision, recall, and F1-scores were calculated for each class under the binary-, four-, and five-class models, as summarized in Supplementary Tables S4–S6. In addition, macro- and micro-averaged performance metrics were computed for the four- and five-class models to evaluate the impact of class imbalance and to differentiate overall predictive performance from class-wise sensitivity, particularly for sparsely represented caries categories (Table 6). While micro-averaged metrics indicated stable overall performance across both models, macro-averaged metrics revealed substantial performance degradation in minority classes, reflecting the pronounced class imbalance in this pilot dataset.

**Table 6:** Macro- and micro-averaged performance metrics (sensitivity/recall, precision, and F1-score) for the four- and five-class models. NaN (Not a Number) indicates an undefined macro-averaged F1-score resulting from the absence of true positive or predicted instances for one or more classes.

Model type	Sensitivity/recall (macro)	Precision (macro)	F1-score (macro)	Sensitivity/recall (micro)	Precision (micro)	F1-score (micro)
Four-class	0.555	0.668	NaN	0.772	0.772	0.772
Five-class	0.504	0.654	NaN	0.813	0.813	0.813

## Discussion

In this pilot study, we explored the feasibility of applying AI algorithms to support the detection and staging of dental caries using self-captured smartphone images. The models consistently demonstrated a high diagnostic accuracy (ranging from 93.6% to 94.4%) across the binary, four-class, and five-class schemes, although the performance declined as classification tasks became more granular. Overall, these findings highlight the potential of smartphone-based AI approaches as adjunctive tools to facilitate patient-driven screening and preliminary assessment of dental caries.

As this study relied exclusively on visual assessment of smartphone photographs, efforts were made to minimize examiner subjectivity through calibration and standardized training. Intra-examiner reliability was high, with weighted Cohen's kappa values ranging from 0.765 to 0.952, indicating substantial to almost perfect agreement. Higher consistency was observed among examiners with greater clinical experience, suggesting that expert judgment contributes to more reproducible visual caries detection and supports the inclusion of senior clinician annotations when establishing ground-truth datasets for AI training. In contrast, inter-examiner agreement was low to moderate, with Fleiss' kappa improving only slightly from κ = 0.358 to κ = 0.406 across repeated assessments, consistent with previous reports showing greater variability in smartphone-based evaluations compared with clinically acquired images (17, 30). Despite calibration using ICDAS-II and ADA CCS criteria, achieving complete consensus remained challenging, particularly when distinguishing between sound and initial lesions or between initial-1 and initial-2 categories, where subtle enamel changes were often influenced by lighting conditions and photographic artifacts. These findings highlight the inherent subjectivity of early-stage visual caries assessment and underscore the need for more refined image acquisition protocols, automated quality control, and AI-assisted diagnostic support to improve consistency; nevertheless, the observed improvement across repeated assessments demonstrates the value of iterative calibration in strengthening the reliability of consensus ground-truth labels.

Beyond examiner-level variability, the difficulty in discriminating closely related caries stages reflects a cascade of compounding factors. Early carious lesions represent a diagnostic continuum rather than discrete categories, and their visual differentiation relies on subtle enamel changes that remain ambiguous even among experienced clinicians. This inherent clinical diagnostic uncertainty was directly reflected in the low inter-examiner agreement observed in this study (Fleiss’ κ = 0.358–0.406), particularly for early-stage lesions. As ground-truth labels were derived from expert consensus on smartphone photographs rather than from standardized clinical examination, label reliability for Initial-1 lesions was inherently limited, as evidenced by the lowest per-class *κ* values for this category (mean κ = 0.58 ± 0.15; Table 4). When trained on labels that carry diagnostic uncertainty, the model inevitably inherits this ambiguity, resulting in increased misclassification between adjacent early-stage categories, as observed in the five-class confusion matrix (Figure 6C). Addressing this limitation will require not only expanded datasets with more balanced class representation, but also more standardized image acquisition protocols and refined annotation strategies to improve ground-truth label reliability for early-stage lesions.

Several previous studies have explored visual caries diagnosis using tooth images. Yoon et al. (17) trained their model on a large dataset of dentist-captured digital camera images but focused exclusively on moderate-to-severe lesions, thereby excluding early-stage caries. In contrast, the present study utilized patient self-taken smartphone photographs and further subdivided initial lesions into two categories (initial-1 and initial-2), directly addressing the challenge of early detection. Similarly, Pandey et al. (30) evaluated smartphone images and reported strong diagnostic performance; however, their analysis was limited to a binary classification without considering lesion severity. Other groups, including Adnan etal (31). and Ahmed et al. (32) achieved favorable performance with advanced CNN- or transformer-based models, but their analyses were confined to clinically acquired images and lacked stage-wise lesion classification. Collectively, these distinctions highlight that the present study uniquely validates stage-specific caries detection in a self-screening context, establishing its novelty relative to prior approaches.

Although the present study demonstrated strong diagnostic performance across all classification schemes (sensitivity ≥86%, specificity >97%, accuracy >93%, and F1-scores >88%), mAP values were comparatively lower (0.51–0.65 at an IoU of 0.5) and declined with increasing classification granularity. This discrepancy reflects inherent challenges of smartphone-based, self-acquired intraoral imaging, including variability in angulation, focus, lighting conditions, and device characteristics, which can obscure subtle enamel changes, particularly in early-stage lesions. Unlike clinically acquired images used in previous studies (17, 31, 32), such variability is difficult to control in self-screening settings, and the inability to consistently reproduce conditions such as air-drying further limits early caries visualization. Among the lesion categories evaluated, ICDAS code 1 lesions are most susceptible to this constraint, which was reflected in the lowest per-class κ values for the Initial-1 category (mean κ = 0.58 ± 0.15; Table 4) and the greatest misclassification between Initial-1 and adjacent categories in the five-class model (Figure 6C). This limitation is particularly fundamental for Initial-1 classification, as ICDAS code 1 lesions are defined by visual changes detectable only after prolonged air-drying under controlled conditions — a requirement inherently unmet in self-acquired smartphone imaging. The inclusion of this category therefore reflects the exploratory intent of this pilot study rather than a claim of reliable clinical detection, and future studies should incorporate standardized drying protocols to improve early-stage lesion identification. As a threshold-independent metric sensitive to class imbalance, mAP is known to be influenced by skewed data distributions (33). Accordingly, in the present study, mAP was strongly affected by the predominance of non-caries images (>80%) and the sparse representation of early and moderate lesions, thereby limiting stable learning for minority classes. The modest mAP values further reflect the inherent difficulty of precisely delineating early-stage lesions such as Initial-1, which lack well-defined boundaries; this ambiguity affects bounding box accuracy from the annotation stage onward, as imprecise ground-truth delineation propagates into model predictions and reduces IoU-based detection scores. Furthermore, the high accuracy and specificity observed across all classification schemes are likely influenced by this class imbalance, which may inflate these aggregate metrics while obscuring limited sensitivity for minority caries categories. The discrepancy between micro- and macro-averaged metrics (Table 6) further reflects this limitation, underscoring that overall performance statistics should be interpreted with caution in the context of severe class imbalance. Unlike accuracy and specificity, which are insensitive to localization errors, mAP jointly penalizes both misclassification and imprecise lesion localization, making it a more stringent indicator of detection performance, particularly for subtle and minority caries categories. Nevertheless, the observed performance suggests that smartphone-based models may serve as adjunctive tools for stage-aware caries screening and preliminary self-assessment by patients.

Consistent with this interpretation, the macro-averaged indicators (Table 6) were substantially lower than the corresponding micro-averaged metrics. This discrepancy helps explain the relatively modest mAP values observed in this study, as macro-averaged metrics are sensitive to class-wise detection performance and therefore reflect reduced sensitivity for early or infrequent lesion categories. While the models achieved high overall accuracy, performance for minority classes was disproportionately affected by severe class imbalance, which is not fully captured by aggregate metrics alone. By contrasting macro- and micro-averaged results, the present findings illustrate how class imbalance can obscure limitations in minority-class detection and contribute to lower mAP in multi-class settings. These results point to the importance of expanding the dataset and applying class-aware augmentation strategies to improve learning for underrepresented caries categories in future work.

Several methodological limitations of this pilot study warrant explicit acknowledgment. First, the small sample size of 70 participants recruited from a single institution limits the generalizability of the findings to broader populations. Second, image acquisition was restricted to mandibular occlusal and labial surfaces, limiting the representativeness of the dataset with respect to the full intraoral dentition. Third, ground-truth labels were based exclusively on expert visual assessment of smartphone photographs without an independent clinical reference standard, introducing inherent uncertainty particularly for early-stage lesions. This limitation is especially relevant for Initial-1 classification, as ICDAS code 1 lesions require air-drying under controlled conditions for reliable visual detection — conditions that could not be replicated in the self-acquired imaging setting of this study. Fourth, the pronounced class imbalance, with sound surfaces comprising more than 80% of all instances, limited stable learning for minority caries categories and may have inflated aggregate metrics. Fifth, variability in smartphone device characteristics and ambient lighting conditions could not be fully eliminated despite standardized protocols, potentially introducing inconsistencies in image quality. In addition, potential sources of bias should be considered. Selection bias may have been introduced, as participants were recruited from a single institution and were required to be capable of following smartphone imaging instructions, thereby excluding individuals with limited digital literacy or physical difficulties in self-capture. Volunteer bias cannot be excluded, as individuals who agreed to participate may have been more health-conscious or more comfortable with smartphone technology than the general population. Furthermore, verification bias may have been present, as ground-truth labels were derived from expert visual assessment of smartphone photographs rather than from an independent clinical reference standard. These limitations underscore the exploratory nature of this pilot study and highlight the need for larger, multi-institutional datasets, expanded image coverage, and incorporation of clinical reference standards in future work.

The clinical implications of this study extend beyond the laboratory setting. From a public health perspective, smartphone-based AI caries detection has particular relevance as a remote screening tool in teledentistry, where patients can self-acquire intraoral images outside the clinical setting and have them assessed remotely by dental professionals or AI-assisted platforms. This approach may be especially beneficial for populations with limited access to dental care due to geographical, economic, or workforce-related barriers, enabling early risk identification and timely referral prior to an in-person visit. Furthermore, by providing patients with objective, stage-specific feedback on their caries status, such tools have the potential to enhance patient engagement, support preventive behavior, and facilitate shared decision-making in dental care. While the present study represents a preliminary feasibility evaluation, these findings suggest that smartphone-based deep learning models could serve as a viable adjunct to conventional clinical workflows in the context of digital and remote oral healthcare delivery.

This study was designed to evaluate the feasibility of AI-based caries detection in a non-clinical, patient-driven setting; therefore, auxiliary diagnostic modalities such as bitewing radiographs and chairside clinical examinations were intentionally excluded. Rather than replicating conventional diagnostic workflows, the objective was to determine whether visual patterns captured solely from patient-acquired smartphone images could support remote screening or preliminary risk stratification as an adjunct to conventional clinical evaluation, without an in-person dental visit. Future studies should expand image coverage, refine imaging protocols, incorporate clinical reference standards, and address class imbalance to enhance model generalizability and support clinical translation.

## Conclusions

In conclusion, this pilot study demonstrated that smartphone-based deep learning models can support the detection and classification of dental caries across multiple severity levels with an overall accuracy exceeding 93%. By leveraging expert consensus annotations and the state-of-the-art YOLOv6-L6 framework, the preliminary model exhibited robust performance across binary and multi-class tasks. This approach highlights the potential of smartphone-based deep learning as an adjunctive tool for patient self-monitoring and preliminary screening, facilitating early awareness and risk assessment prior to clinical evaluation.

## Data Availability

The raw data supporting the conclusions of this article will be made available by the authors, without undue reservation.
