# Supplementary material for: Feasibility of multi-class dental caries detection using deep learning–based smartphone images: a pilot prospective study
Source: Front Oral Health. 2026 May 8;7:1805448. doi: 10.3389/froh.2026.1805448 (PMC13194414; doi:10.3389/froh.2026.1805448)
Supplement: Supplementary file 1 [file Table1.docx]

**Table S1.** Smartphone acquisition details and imaging conditions.

| Parameter | Description |  |
| --- | --- | --- |
| Number of participants | 70 adults | |
| Images per participant | Six intraoral photographs following standardized imaging guidelines | |
| Image views | (1–3) Frontal, right lateral, and left lateral views with teeth in occlusion and lips parted to capture labial surfaces; (4–6) Anterior, right, and left mandibular views with mouth open to expose occlusal surfaces | |
| Tooth-surface instances | 4,932 | |
| Device type | 23 Android / 47 iOS smartphones (self-reported) | |
| Camera type | Front-facing camera (selfie mode) | |
| Flash | Not used | |
| Lighting condition | Natural daylight preferred; flash and external light sources prohibited; camera brightness set to approximately 60% | |
| Zoom | Not used | |
| Camera-to-tooth distance | Approximately 20–30 cm | |
| Image resolution | 1920×1080 or higher | |
| Shooting guidance | Participants used the grid function to center the image, maintained a consistent distance, and aligned the camera lens parallel to the tooth surface | |
| Pre-capture preparation | Participants were instructed to swallow saliva and remove debris prior to capture; camera lens was cleaned before use | |
| Patient guidance materials | Standardized written protocol with reference photographs and visual framing guides illustrating recommended image composition ratio provided for each of the six views | |
| Image retake policy | Participants were contacted to retake unclear images affected by blur, glare, or saliva | |
| Failed-image rate | Not calculated (unclear images were replaced through retakes) | |
| Anonymization | All images were anonymized and stored without identifying information | |

**Table S2.** STARD-AI checklist adherence.

| Item | STARD-AI item (abbreviated) | Reported (Yes/No) | Page |
| --- | --- | --- | --- |
| 1 | AI-based diagnostic accuracy study identified in title/abstract | Yes | p. 1 |
| 2 | Structured abstract with performance metrics | Yes | p. 1 |
| 3 | Clinical background and intended use of AI described | Yes | p. 2-3 |
| 4 | Study objectives stated | Yes | p. 4 |
| 5 | Study design (retrospective/prospective) reported | Yes | p. 4 |
| 6 | Ethical approval or exemption reported | Yes | p. 4 |
| 7 | Inclusion and exclusion criteria defined | Yes | p. 4-5 |
| 8 | Tooth-level or patient-level unit specified | Yes | p. 4 |
| 9 | Study setting and data collection period reported | Yes | p. 5 |
| 10 | Data source and image acquisition described | Yes | p. 5-6 |
| 11 | Reference standard (e.g., ICDAS) clearly defined | Yes | p. 6 |
| 12 | AI model description (inputs/outputs) provided | Yes | p. 7-9 |
| 13 | Dataset split (train/validation/test) reported | Yes | p. 6-7 |
| 14 | Diagnostic performance metrics defined | Yes | p. 9 |
| 15 | Handling of missing or uncertain data described | Yes | p. 6 |
| 16 | Distribution of disease severity reported | Yes | p. 10 |
| 17 | Confusion matrix or equivalent results provided | Yes | p. 17 |
| 18 | Study limitations discussed | Yes | p. 22 |
| 19 | Clinical implications and intended role of AI discussed | Yes | p. 17 |
| 20 | Data availability and funding disclosed | Yes | p. 21-22 |

**Table S3.** Training configuration for the YOLov6-L6-based dental caries detection model.

| Parameter | Configuration |  |
| --- | --- | --- |
| Input size | 1280 × 1280 | |
| Normalization / Color space | Pixel normalization (0-1 range), RGB color space | |
| Data augmentation probability | 0.5 | |
| Optimizer | SGD (momentum = 0.937, weight decay = 0.0005) | |
| Learning rate scheduler | Cosine annealing with warm-up and EMA | |
| Batch size | 8 (2 per GPU batch) | |
| Epochs | 24 | |
| Early stopping | Not applied | |
| Dropout | None (implicit regularization via data augmentation) | |
| Random seed | Not specified | |
| Loss function | VFL + SloU Loss | |
| Label assignment | Task-Aligned Learning (TAL) | |
| Backbone / Neck | EfficientRep Backbone / Rep-PAN Neck | |
| Detection head | Efficient Decoupled Head | |
| Framework | YOLOv6-L6 | |

SGD: stochastic gradient descent, VFL: VariFocal Loss, SloU: SCYLLA Intersection over Union, TAL: Task-Aligned Learning

**Table S4.** Class-wise performance metrics (confidence threshold, sensitivity/recall, precision, F1-score, AP@0.5:0.95, and AP@0.5) for the binary classification model.

| Class name | Confidence threshold | Sensitivity/  Recall | Precision | F1-score | AP  @(0.5:0.95) | AP@0.5 |
| --- | --- | --- | --- | --- | --- | --- |
| None | 0.52 | 0.89 | 0.86 | 0.89 | 70.8 | 85.9 |
| Initial | 0.84 | 0.44 | 0.81 | 0.57 | 39.0 | 45.5 |

**Table S5.** Class-wise performance metrics (confidence threshold, sensitivity/recall, precision, F1-score, AP@0.5:0.95, and AP@0.5) for the four-class model.

| Class name | Confidence threshold | Sensitivity/  Recall | Precision | F1-score | AP  @(0.5:0.95) | AP@0.5 |
| --- | --- | --- | --- | --- | --- | --- |
| None | 0.51 | 0.82 | 0.91 | 0.86 | 69.1 | 85.1 |
| Initial | 0.72 | 0.32 | 0.44 | 0.37 | 18.9 | 23.9 |
| Moderate | 0.58 | 0.08 | 0.37 | 0.13 | 5.2 | 5.8 |
| Advanced | 0.86 | 1.00 | 0.95 | 0.98 | 87.0 | 97.6 |

**Table S6.** Class-wise performance metrics (confidence threshold, sensitivity/recall, precision, F1-score, AP@0.5:0.95, and AP@0.5) for the five-class model.

| Class name | Confidence threshold | Sensitivity/  Recall | Precision | F1-score | AP  @(0.5:0.95) | AP@0.5 |
| --- | --- | --- | --- | --- | --- | --- |
| None | 0.60 | 0.87 | 0.90 | 0.89 | 72.6 | 87.5 |
| Initial-1 | 0.75 | 0.16 | 0.30 | 0.21 | 9.5 | 10.9 |
| Initial-2 | 0.61 | 0.27 | 0.59 | 0.37 | 20.0 | 22.2 |
| Moderate | 0.30 | 0.33 | 0.69 | 0.45 | 15.2 | 29.5 |
| Advanced | 0.72 | 0.89 | 0.79 | 0.84 | 73.3 | 89.9 |
